# Heterogeneous photochemical reaction enabled by an ultrasonic microreactor†

**DOI:** 10.1039/d3re00154g

**Published:** 2023-04-24

**Authors:** Aniket P. Udepurkar, Kakasaheb Y. Nandiwale, Klavs F. Jensen, Simon Kuhn

**Affiliations:** a KU Leuven, Department of Chemical Engineering Celestijnenlaan 200F 3001 Leuven Belgium simon.kuhn@kuleuven.be; b Department of Chemical Engineering, Massachusetts Institute of Technology 77 Massachusetts Avenue Cambridge Massachusetts 02139 USA kfjensen@mit.edu

## Abstract

The presence of solids as starting reagents/reactants or products in flow photochemical reactions can lead to reactor clogging and yield reduction from side reactions. We address this limitation with a new ultrasonic microreactor for continuous solid-laden photochemical reactions. The ultrasonic photochemical microreactor is characterized by the liquid and solid residence time distribution (RTD) and the absorbed photon flux in the reactor *via* chemical actinometry. The solid-handling capability of the ultrasonic photochemical microreactor is demonstrated with a silyl radical-mediated metallaphotoredox cross-electrophile coupling with a solid base as a reagent.

## Abstract 2

A novel ultrasonic microreactor is demonstrated for the heterogeneous silyl radical-mediated metallaphotoredox cross-electrophile coupling.

## Graphical abstract

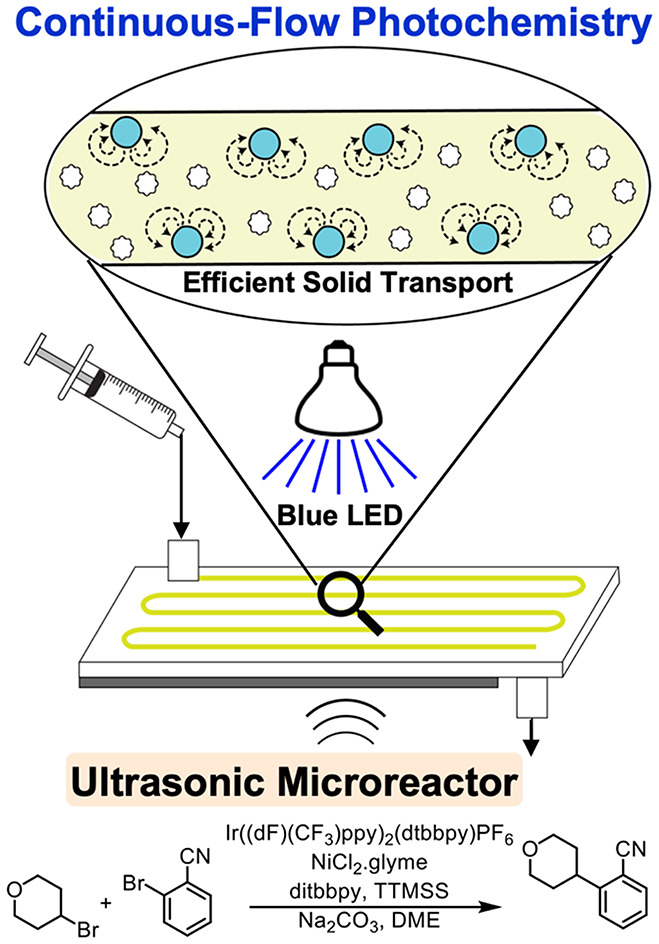

## Introduction

As flow chemistry is gaining prominence, microreactors are increasingly employed due to their high surface-to-volume ratio offering better heat and mass transfer, safer operating conditions, and a smaller footprint than batch reactors.^1–7^ Moreover, microreactors offer a small penetration depth leading to improved photon flux, making them an excellent choice for photochemical reactions in flow.^8–10^ However, solids present as reagents or products during the reaction may lead to microreactor fouling and clogging.^11–13^ The integration of ultrasound with a microreactor has proven to be effective in mitigating fouling and clogging in the microchannels.^14–16^ The cavitation bubbles generated by the ultrasound actuation oscillate and collapse, leading to microstreaming, micro-jets, and vortices in the microchannels.^17^ These physical effects can resuspend solids and prevent agglomeration and bridging of the solids.^17^

Ultrasonic microreactors have been utilized in solid-generating reactions to prevent clogging by actuating low-frequency and high-frequency ultrasound.^18–21^ Pulsed ultrasound actuation has also proven equally beneficial in preventing clogging in the microreactors.^22,23^ Delacour *et al.* successfully scaled up an ultrasonic flow reactor for solid handling with increased productivity by two orders of magnitude at a remarkably low load power per volume (0.48 W mL^−1^).^24^ Ultrasonic microreactors have also been used for organic synthesis having solid catalysts and reagents.^25–28^ Horie *et al.* utilized a tubular microreactor placed in an ultrasonic bath with a gas–liquid segmented flow for the photodimerization of maleic anhydride having solid byproducts.^29^ The reactor could be operated continuously for 16 hours without clogging and obtained better product quality and improved conversion than the batch reactor.^29^ Recently, Dong *et al.* utilized an ultrasonic millireactor for a gas–solid–liquid photocatalytic reaction.^30^ Ultrasound enabled resuspension of TiO_2_ nanoparticles to prevent clogging with improved reaction conversion.^30^ Thus, ultrasonication has proven to be an effective tool for solid handling with improved mixing in microreactors.^22–25,31,32^

The previous studies on the utility of ultrasonic microreactors for solid-catalyzed reactions and by-products focused on particles in size range of 0.5–5 μm.^25,26,28–30^ In addition, the ultrasonic microreactors are operated at a relatively high ultrasonic power of 20–60 W. In this study, we utilize particles with a size range of 10–50 μm with a short settling time of a few seconds (see Table S4†). The large particle size and fast settling time pose challenges for solid handling and fouling in microchannels. Our ultrasonic microreactor tackles this challenge while working at a low ultrasonic power of 5 W. In addition, no reports exist on the solid residence time distribution (RTD) in ultrasonic microreactors. The solid RTD can provide valuable insights into the solid transport in the microreactor and aid in detecting possible accumulation of solids in the microreactor. Finally, we use silyl radical-mediated metallaphotoredox cross-electrophile coupling with a solid base as a case study to demonstrate the handling of solids in flow.

## Design, fabrication, and characterization of ultrasonic microreactor

### Design and fabrication of ultrasonic microreactor

**Fig. 1:**
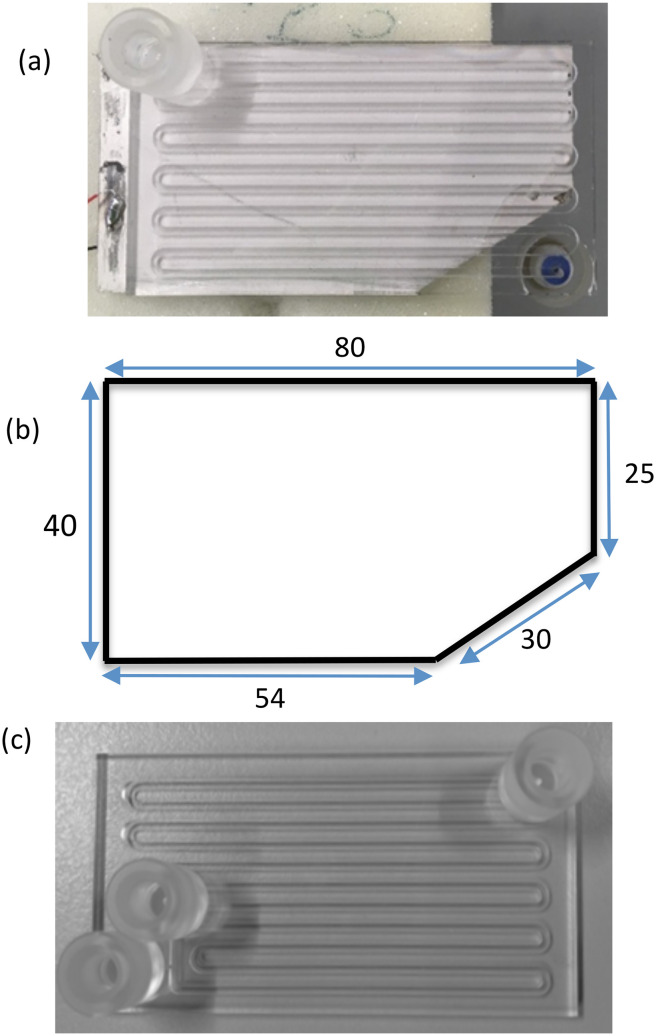
(a) Glass reactor R1 bonded to piezoelectric plate transducer. The inlet and outlet of the reactor are on two opposite faces of reactor, (b) dimensions of piezoelectric plate transducer for the reactor R1, and (c) glass reactor R2.

The glass reactor for photochemical reaction (R1) (Little Things Factory, Germany) was fabricated by sandwiching a borosilicate glass layer of 1.2 mm thickness between two glass layers of 1 mm (Fig. 1(a)). A serpentine channel with a cross-section of 1.2 × 1.2 mm^2^ and a length of 76 cm was etched in the middle layer and coated with a hydrophobic silane coating. The total volume of the reactor is 1.1 mL. Both an inlet and outlet ports were made up of glass and with ¼′′ UNF 28 threads bonded onto the two opposite faces of the reactor connecting the serpentine channel *via* a bore of diameter 1.2 mm on the glass plate. The reactor was placed horizontally with the outlet flow in the direction of gravity to ensure that solids easily exited the reactor. The glass reactor (R2) used for the RTD study was fabricated similarly with a hydrophobic channel of 1.2 × 1.2 mm^2^ cross-section and a volume of 1.2 mL (Fig. 1(c)). This reactor had two inlet ports and an outlet port on the same face of the reactor.

A piezoelectric plate transducer (Pz-26, Ferroperm, Denmark) was bonded to the reactor using epoxy glue (Epotek-301, USA) to actuate ultrasound. The piezoelectric plate of R1 was cut to the dimension as shown in Fig. 1(b) and had a thickness of 1.67 mm. The piezoelectric plate dimensions for R2 are 80 × 40 × 1.67 mm^3^. The resonance frequency of 48 kHz for both reactors was determined using an impedance analyzer (16777k, SinePhase). Ultrasound was actuated by connecting the reactor to an amplifier (2100L, E&I) and waveform generator (SDG1025, Siglent) to generate a sinusoidal waveform with the frequency of 48 kHz and a selected amplitude to get the desired load power. The ¼-28 Flat-Bottom fittings and ferrules for connecting PFA tubing of 1/16′′ OD were used at the inlet and outlet ports. PFA tubing of 1/16′′ OD was used to deliver the reagents (liquid/suspension) to the reactor.

### Liquid residence time distribution

**Fig. 2:**
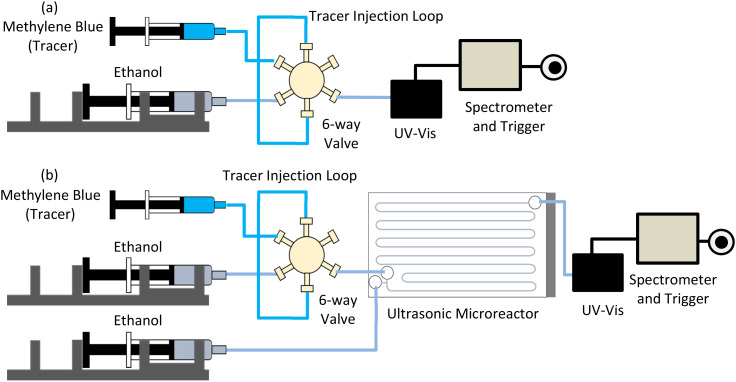
(a) Schematic of liquid RTD measurement at inlet: six-way valve outlet is connected to UV-vis flow cell, (b) schematic of liquid RTD measurements of reactor at outlet configuration: the reactor is connected between the six-way valve and the UV-vis flow cell.

The liquid residence time distribution (RTD) of reactor R2 was obtained using a pulse injection technique. Ethanol was used as the continuous phase, and methylene blue was dissolved in ethanol (0.3 mg mL^−1^) as a tracer. A manual six-way valve (V-451, IDEX) with a sample loop was employed to inject the tracer into the reactor. In-line UV-vis spectroscopy was used to obtain the concentration profile of the tracer. The relationship between the concentration and the absorbance for methylene blue was linear for the desired concentration range used to measure the liquid RTD (Fig. S2†). The spectrometer was connected to an in-house trigger. As soon as the tracer was injected, the trigger was switched to the ON position and data collection started using Avantes software. To measure the inlet profile for the tracer injection, the 6-way valve was directly connected to the UV-vis flow cell (Fig. 2(a)). The outlet RTD measurement was obtained by placing the reactor between the six-way valve and UV-vis (Fig. 2(b)). The liquid RTD was obtained for the sonicated condition (frequency: 48 kHz, power: 5 W) and silent condition for the residence time of 11 minutes. On blocking one of the two inlets of the reactor, a small amount of the tracer would enter the blocked inlet channel during the silent condition measurements and release slowly, leading to a long tail and a significant deviation from the calculated residence time. To counter this backflow, a gentle stream of ethanol was added to the second inlet at a 0.1 μL min^−1^ flow rate for all the RTD measurements. Ethanol was delivered to the reactor using a syringe pump (Fusion 200, Chemyx). The reactor was placed on a heat sink during sonication, and the temperature was regulated using a cooling fan. Ethanol was degassed before the measurements to avoid the formation of large gas slugs during the sonication. The inlet and outlet measurements were repeated three times for both silent and sonicated conditions (details in the ESI†).

### Solid residence time distribution

**Fig. 3:**
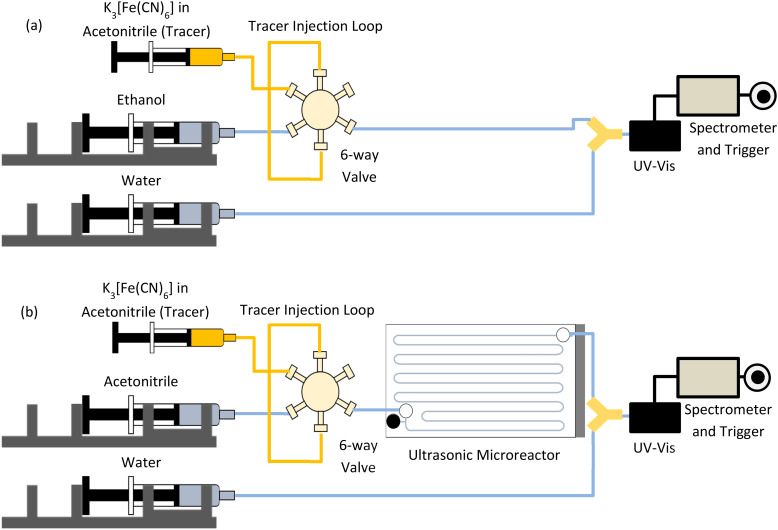
Schematic of the inlet configuration for solid RTD measurement. (a) The outlet of the six-way valve is connected to a Y-junction along with water to dissolve solids before they reach the UV-vis flow cell, (b) schematic of the solid RTD measurements of the reactor at outlet configuration.

Solid RTD experiments were carried out using acetonitrile as the continuous phase and potassium ferricyanide (K_3_[Fe(CN)_6_]) with a mean diameter (*d*_50_) of 22.5 μm as a solid tracer. As for the liquid RTD experiments, a manual six-way valve was employed to inject a pulse of solid tracer particles to obtain the solid RTD. The (K_3_[Fe(CN)_6_]) particles were milled and then sieved with sieve no. 200 to obtain the particles for solid RTD measurements. The particle size of the tracer particles was measured by laser diffraction (Malvern Mastersizer 3000) (Fig. S4†). A suspension of K_3_[Fe(CN)_6_] in acetonitrile (9 mg mL^−1^) was prepared to measure the solid RTD. The concentration *vs.* absorbance was linear for the concentration range of solid investigated in the solid RTD (Fig. S3†). K_3_[Fe(CN)_6_] suspension was injected into the sample loop just before injecting the particles in the reactor to avoid settling of particles in the sample loop. The reactor inlet tubing was placed vertically to ensure efficient solids delivery to the reactor. The second inlet port of the reactor was closed during the measurements. At the exit of the reactor, a Y-junction was employed to mix the incoming solid-acetonitrile stream with water to dissolve the solid particles. The inlet measurements were carried out by connecting the six-way valve directly to the Y-junction (Fig. 3(a)). The solid RTD outlet measurements were carried out by connecting the reactor between the six-way valve and UV-vis line, as shown in Fig. 3(b). The solid RTD measurements were performed for the sonicated case. During the sonication, the reactor was placed on a heat sink and cooled with a cooling fan to control the temperature. Acetonitrile was degassed for the solid RTD measurements.

## Results and discussion

The solid handling capability of the reactor was first tested to find suitable solid loading and ultrasound operating parameters. A suspension of sodium carbonate (Na_2_CO_3_) in ethanol was prepared for concentrations of 2.2 mg mL^−1^ and 4.4 mg mL^−1^. The suspension was introduced in a syringe and a magnetic stirring plate was placed close to it to continuously stir the suspension during delivery. The pump and inlet tubing of short length were placed vertically to avoid solids settling down in the tubing and ensure a steady delivery of the suspension to the reactor. The solid transport in the reactor was evaluated for a residence time of 10 minutes and 20 minutes. The reactor channel was visually observed to detect any accumulation of solids and clogging of the channels.

For the solid loading of 2.2 mg mL^−1^, no solid accumulation was observed in the channels during an operation window of 60 minutes for the residence times of 10 minutes and 20 minutes. Moreover, on increasing the solid loading to 4.4 mg mL^−1^ the solids were transported with ease with no visible accumulation in the channels for a residence time of 10 minutes for an operation window of 60 minutes. However, for the residence time of 20 minutes and solid loading of 4.4 mg mL^−1^, the Na_2_CO_3_ particles were seen to accumulate in the channel after 40 minutes. Channel fouling can pose a risk of clogging during long-term operation and lower the reaction yield. Based on the results of the preliminary experiments, a solid loading of 4.4 mg mL^−1^ with a residence time of 11 minutes was chosen to aim for a high yield in the photochemical reaction.

### Liquid and solid residence time distribution

The liquid RTD was determined to quantify the experimental liquid residence time and characterize the mixing characteristics of the ultrasonic microreactor. In a microreactor, the laminar flow leads to large axial dispersion and a broad residence time distribution, which could result in a low conversion or possible side reactions.^1^ The cavitation bubbles generated in the microchannel on sonication can increase mixing and lead to a narrower RTD.^32^ The axial dispersion model was used to investigate the axial dispersion during the silent and the sonicated case.^8,33,34^ The RTD calculations are further elaborated in the ESI.†

**Fig. 4:**
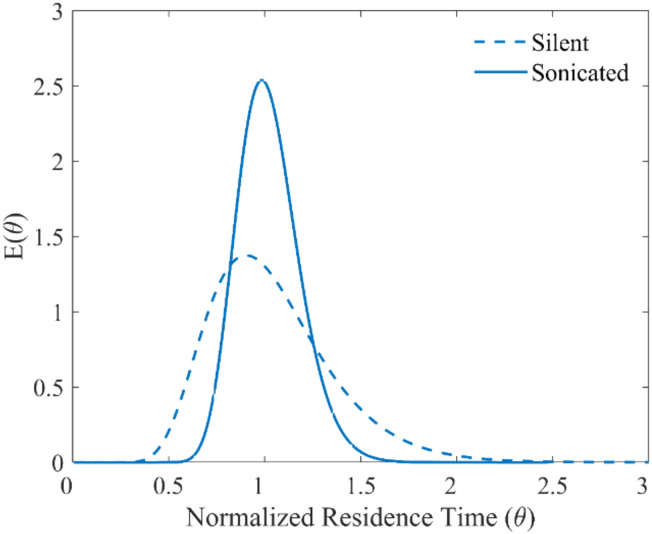
Liquid RTD for the ultrasonic microreactor for the silent and sonicated case (residence time 11 min).

Based on the preliminary results of solid handling in the ultrasonic microreactor, a theoretical residence time of 11 minutes was chosen to study the RTD in the reactor for silent and sonicated conditions. For the silent case, the mean experimental residence time was 637.5 seconds, and the vessel dispersion number was 0.048. The vessel dispersion number defines the extent of axial dispersion in the reactor. The higher the vessel dispersion number, the greater the axial dispersion and therefore, the wider the RTD. When ultrasound was actuated at the frequency of 48 kHz and power of 5 W, the vessel dispersion number decreased to 0.012 while the mean residence time was 639.3 seconds. Thus, the introduction of ultrasound decreased the axial dispersion leading to a narrower RTD of the reactor, as seen in Fig. 4, while the residence time is not significantly influenced. The microstreaming due to the cavitation bubbles in the microchannel improved the radial mixing.^32^

Dong *et al.* investigated the application of an ultrasonic milli-reactor for photocatalytic oxidation using TiO_2_ nanoparticles and agglomerates.^30^ They reported a settling time of 105 seconds in the tube of diameter 2 mm. They reported a yield of 20–60% for the non-sonicated conditions. The micrometer-sized K_3_[Fe(CN)_6_] and Na_2_CO_3_ particles have a theoretical settling time of 2.5 seconds and 1.4 seconds respectively, which is two orders of magnitude smaller than the settling time for the TiO_2_ nanoparticles (see Table S4†). Zhang *et al.* reported a meager yield of 3% for the silyl radical-mediated metallaphotoredox reaction in absence of a base.^35^ The settling of the solid base particles would lead to a depletion or absence of the base in the microchannel resulting in a low yield. The low settling time of the solid particles of a few seconds coupled with a long residence time of 11 minutes also poses a risk of fouling and clogging in the microchannel. Thus, the efficient transport of the solids is of prime importance for the photochemical reaction of choice. Along with a narrow liquid residence time distribution, a narrow solid residence time distribution is desired to maintain the stoichiometric ratio for the reaction and avoid an accumulation of solids in the channel over time. For characterizing the solid residence time distribution in the reactor a pulse of K_3_[Fe(CN)_6_] suspended in acetonitrile was injected into the reactor. The axial dispersion model was utilized to study the solid transport through the reactor.

**Fig. 5:**
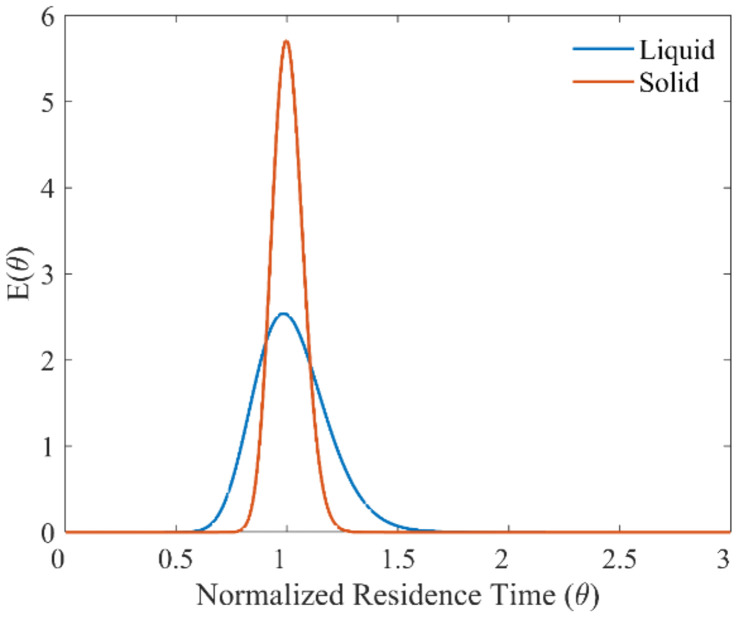
Solid and liquid (sonicated case) RTD for the ultrasonic microreactor (residence time 11 min).

For the applied ultrasound frequency of 48 kHz and power of 5 W, the experimental residence time obtained was 669.9 seconds. The vessel dispersion number for the solid RTD was 0.002. Comparing the liquid (sonicated case) and solid RTD in Fig. 5, it can be seen that solids have narrower residence time distribution. From the solid RTD, it is evident that the ultrasonic microreactor is capable of transporting solids while avoiding any local accumulation in the channels.

The liquid and the solid RTD profiles of the ultrasonic microreactor indicate that the reactor is capable of handling solids for the photochemical reaction while maintaining a local stoichiometric ratio during the reaction. The narrow liquid and solid RTD could ensure a stable and high yield for the photochemical reaction of interest.

### Silyl mediated photochemical reaction

**Fig. 6:**
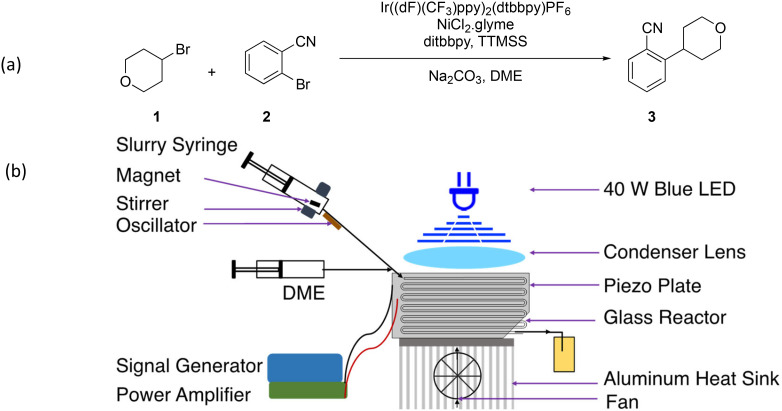
(a) Reaction scheme of silyl mediated photochemical reaction and (b) schematic of the experimental setup for photochemical reaction in an ultrasonic microreactor.

Silyl radical-mediated metallaphotoredox cross-electrophile coupling^35^ (1 and 2) with a solid base (Na_2_CO_3_) of mean diameter (*d*_50_) 21 μm as a reagent was chosen as a case study to demonstrate the solid-handling capability of the ultrasonic microreactor (Fig. 6a). Chemical actinometry of the microreactor was performed at different electrical input powers of 40 W blue LED to measure the absorbed photon flux (Table 1) (details in the ESI†).

**Table 1:** Absorbed photon flux in the microreactor using different LED input power

LED input power (%)	DPA conversion (%)	Absorbed photon flux (mol s^−1^ m^−3^)
5	5.4	0.09
10	8.2	0.14
25	16.4	0.28
50	30.1	0.52
75	49.5	0.87
100	81.8	1.43

The suspension containing the reactants and the solid base was loaded in a stainless-steel syringe with a PTFE-coated magnetic stir bar. Syringe pump 1 (Harvard Apparatus PhD Ultra), was kept at an angle to deliver the suspension. The suspension was stirred by a magnetic tumbler/stirrer (V&P Scientific VP 710D3-4) to keep the suspension uniform (Fig. 6(b)).^36^ In addition, enhanced slurry transport was achieved with an oscillator (Precision Microdrives 306-10H) to the tube between the microreactor and syringe. Syringe pump 2 was utilized to deliver degassed DME, which was used for the microreactor prefilling and clean-up.

The experiment was initialized by prefilling the microreactor with the degassed DME (syringe pump 2). The ultrasonic microreactor was sonicated (frequency: 48 kHz, power: 5 W) and illuminated with the 40 W blue LED during the initialization.

The microreactor was placed on a heat sink, and the temperature was regulated using a cooling fan. The DME flow was stopped, and the slurry flow was started with syringe pump 1. The flow rate was set to 0.1 mL min^−1^ for the residence time of 11 minutes. The FLIR ONE Pro (IR camera) indicated a microreactor temperature of 32 °C during the operation. The photochemical reaction in the ultrasonic microreactor was carried out for 3 hours after the steady state without any clogging issues. The sample at the outlet was collected at the steady state (∼3 residence times) and analyzed with HPLC (Agilent 1260). The steady-state sample analyzed by HPLC indicated the formation of 3 in ∼70% yield.

The ultrasonic microreactor we present can handle solids and prevent any fouling or clogging in the microchannel for the operating window (residence time 11 min, solid loading 4.4 mg mL^−1^) chosen for the silyl-mediated photochemical reaction of interest. The liquid and solid RTD reveals that the sonication leads to a narrow residence time distribution and ensures a local stoichiometric ratio for the photochemical reaction. The HANU reactor coupled with oscillatory flow previously reported for enabling the silyl radical-mediated photochemical reaction has a trade-off between the poor solid handling and the broad liquid RTD.^14^ The developed ultrasonic microreactor can be a viable alternative to the HANU reactor owing to its narrow liquid and solid RTD.

The ultrasonic microreactor has a productivity of 0.084 mmol h^−1^, two orders of magnitude lower than the HANU reactor. The lower reactor volume of the microreactor resulted in lower throughput. However, a comparison of the space–time yield (STY) shows that the ultrasonic microreactor achieved a STY of 2.12 × 10^−2^ mol s^−1^ m^−3^, which is in the same order of magnitude as the CSTR cascade and the HANU reactor (Table 2). The photocatalytic space–time yield (PSTY), which takes into account the productivity coupled with the illumination source electrical consumption, reveals that the ultrasonic microreactor fares well in comparison to the CSTR cascade, but it lags behind the HANU reactor. The energy density of the ultrasonic microreactor was 3 × 10^9^ J m^−3^, which is in the same order of magnitude as the milli-reactor demonstrated by Dong *et al.*^30^ (7.7 × 10^9^ J m^−3^) and Delacour *et al.*^24^ (3.6 × 10^9^ J m^−3^) for heterogeneous synthesis in flow. Further efforts to scale up the ultrasonic microreactor and to design an efficient illumination source coupled with the ultrasonic microreactor could increase the throughput and improve the STY and PSTY.

**Table 2:** Overview of the reactors employed for the silyl radical-mediated metallaphotoredox cross-electrophile coupling with a solid base (Na_2_CO_3_)

Reactor	*C* a (mM)	*V* b (ml)	RT^c^ (min)	Yield^d^ (%)	*R* e (mmol h^−1^)	STY^f^ (mol s^−1^ m^−3^)	PSTY^g^ (mol s^−1^ W^−1^ m^−3^)	*P* h (W)	*λ* i (nm)	*Φ* j (mol s^−1^ m^−3^)
CSTR cascade^1^	40	5.3	30	80	0.339	1.78 × 10^−2^	1.5 × 10^−4^	120	440	0.27488^k^
HANU reactor^14^	20	14	30	80	4.480	8.89 × 10^−2^	1.2 × 10^−3^	75	405	2.12
Ultrasonic microreactor	20	1.1	11	70	0.084	2.12 × 10^−2^	5.3 × 10^−4^	40	440	1.43

a Substrate concentration.

b Reactor volume.

c Reactor residence time.

d Reaction yield.

e Productivity.

f Space-time yield.

g Photocatalytic space–time yield.

h Lamp input power.

i Lamp wavelength.

j Absorbed photon flux.

k Absorbed photon flux for measured for one lamp.

## Conclusions

A novel ultrasonic microreactor was developed for continuous solid-laden photochemical reactions. The liquid and solid RTD profiles of the ultrasonic microreactor indicate that the reactor could handle solids for the photochemical reaction and maintain a local stoichiometric ratio during the reaction. The narrow RTD can ensure a stable and high yield for the reaction. The absorbed photon flux in the reactor was obtained with chemical actinometry. Moreover, the case study of silyl radical-mediated metallaphotoredox cross-electrophile coupling with a solid base as a reagent revealed that the ultrasonic microreactor can handle solids in flow.

## Author contributions

Aniket P. Udepurkar: conceptualization, formal analysis, investigation, methodology, software, validation, visualization, and writing – original draft. Kakasaheb Y. Nandiwale: conceptualization, formal analysis, investigation, methodology, software, validation, visualization, and writing – original draft. Klavs F. Jensen: project administration, conceptualization, supervision, resources, investigation, methodology, validation, writing – review & editing. Simon Kuhn: project administration, conceptualization, supervision, resources, investigation, methodology, validation, writing – review & editing.

## Conflicts of interest

There are no conflicts to declare.

## Supplementary Material

RE-008-D3RE00154G-s001

## References

[cit1] Pomberger A., Mo Y. M., Nandiwale K. Y., Schultz V. L., Duvadie R., Robinson R. I., Altirioglu E. I., Jensen K. F. (2019). Org. Process Res. Dev..

[cit2] Dong Z., Delacour C., Mc Carogher K., Udepurkar A. P., Kuhn S. (2020). Materials.

[cit3] Wu K.-J., Kuhn S. (2014). Chim. Oggi - Chem. Today.

[cit4] Zhao S., Yao C., Dong Z., Chen G., Yuan Q. (2020). Particuology.

[cit5] Elvira K. S., i Solvas X. C., Wootton R. C. R., deMello A. J. (2013). Nat. Chem..

[cit6] Fanelli F., Parisi G., Degennaro L., Luisi R. (2017). Beilstein J. Org. Chem..

[cit7] Yao X., Zhang Y., Du L., Liu J., Yao J. (2015). Renewable Sustainable Energy Rev..

[cit8] Roibu A., Van Gerven T., Kuhn S. (2020). ChemPhotoChem.

[cit9] Tiwari D. K., Maurya R. A., Nanubolu J. B. (2016). Chem. – Eur. J..

[cit10] Mizuno K., Nishiyama Y., Ogaki T., Terao K., Ikeda H., Kakiuchi K. (2016). J. Photochem. Photobiol., C.

[cit11] Scheiff F. and Agar D. W., in Micro-Segmented Flow: Applications in Chemistry and Biology, ed. J. M. Köhler and B. P. Cahill, Springer Berlin Heidelberg, Berlin, Heidelberg, 2014, pp. 103–148

[cit12] Chen Y., Sabio J. C., Hartman R. L. (2015). J. Flow Chem..

[cit13] Dressaire E., Sauret A. (2017). Soft Matter.

[cit14] Debrouwer W., Kimpe W., Dangreau R., Huvaere K., Gemoets H. P. L., Mottaghi M., Kuhn S., Van Aken K. (2020). Org. Process Res. Dev..

[cit15] Navarro-Brull F. J., Poveda P., Ruiz-Femenia R., Bonete P., Ramis J., Gómez R. (2014). Green Processes Synth..

[cit16] Sancheti S. V., Gogate P. R. (2017). Ultrason. Sonochem..

[cit17] Horst C. , Gogate P. R. and Pandit A. B., in Modeling of Process Intensification, 2007, pp. 193–277

[cit18] Rivas D. F., Kuhn S. (2016). Top. Curr. Chem..

[cit19] Castro F., Kuhn S., Jensen K., Ferreira A., Rocha F., Vicente A., Teixeira J. A. (2013). Chem. Eng. J..

[cit20] Dong Z., Rivas D. F., Kuhn S. (2019). Lab Chip.

[cit21] Zhang L., Geng M., Teng P., Zhao D., Lu X., Li J.-X. (2012). Ultrason. Sonochem..

[cit22] Delacour C., Lutz C., Kuhn S. (2019). Ultrason. Sonochem..

[cit23] Dong Z., Udepurkar A. P., Kuhn S. (2020). Ultrason. Sonochem..

[cit24] Delacour C., Stephens D. S., Lutz C., Mettin R., Kuhn S. (2020). Org. Process Res. Dev..

[cit25] Hartman R. L., Naber J. R., Zaborenko N., Buchwald S. L., Jensen K. F. (2010). Org. Process Res. Dev..

[cit26] Noël T., Naber J. R., Hartman R. L., McMullen J. P., Jensen K. F., Buchwald S. L. (2011). Chem. Sci..

[cit27] Kuhn S., Noël T., Gu L., Heider P. L., Jensen K. F. (2011). Lab Chip.

[cit28] Dong C., Wang K., Zhang J. S., Luo G. S. (2015). Chem. Eng. Sci..

[cit29] Horie T., Sumino M., Tanaka T., Matsushita Y., Ichimura T., Yoshida J.-I. (2010). Org. Process Res. Dev..

[cit30] Dong Z., Zondag S. D. A., Schmid M., Wen Z., Noël T. (2022). Chem. Eng. J..

[cit31] Katayama E., Togashi S., Endo Y. (2010). J. Chem. Eng. Jpn..

[cit32] Dong Z., Zhao S., Zhang Y., Yao C., Yuan Q., Chen G. (2017). AIChE J..

[cit33] Danckwerts P. V. (1953). Chem. Eng. Sci..

[cit34] Levenspiel O. (1999). Ind. Eng. Chem. Res..

[cit35] Zhang P., Le C. C., MacMillan D. W. C. (2016). J. Am. Chem. Soc..

[cit36] Nandiwale K. Y., Hart T., Zahrt A. F., Nambiar A. M. K., Mahesh P. T., Mo Y. M., Nieves-Remacha M. J., Johnson M. D., Garcia-Losada P., Mateos C., Rincon J. A., Jensen K. F. (2022). React. Chem. Eng..

