# Supplementary material for: Heterogeneous photochemical reaction enabled by an ultrasonic microreactor
Source: React Chem Eng. 2023 Apr 24;8(8):1930–6. doi: 10.1039/d3re00154g (PMC10388398; doi:10.1039/d3re00154g)
Supplement: RE-008-D3RE00154G-s001 [file RE-008-D3RE00154G-s001.pdf]

Electronic Supplementary Material (ESI) for Reaction Chemistry & Engineering.
This journal is © The Royal Society of Chemistry 2023

Supplementary Information

Heterogeneous Photochemical Reaction enabled by an Ultrasonic Microreactor

Aniket P. Udepurkar,‡[a] Kakasaheb Y. Nandiwale,‡[b] Klavs F. Jensen,*[b] and Simon Kuhn*[a]

[a]KU Leuven, Department of Chemical Engineering, Celestijnenlaan 200F, 3001 Leuven, Belgium.

[b]Department of Chemical Engineering, Massachusetts Institute of Technology, 77 Massachusetts Avenue, Cambridge, Massachusetts 02139, United States.

‡These authors contributed equally to this work.

*Correspondence to:

kfjensen@mit.edu

simon.kuhn@kuleuven.be

Table of Contents

Ultrasonic Microreactor Admittance Curve	3
Liquid Residence Time Distribution	3
Solid Residence Time Distribution	6
Chemical Actinometry	9
Silyl Mediated Photochemical Reaction	9

Ultrasonic Microreactor Admittance Curve

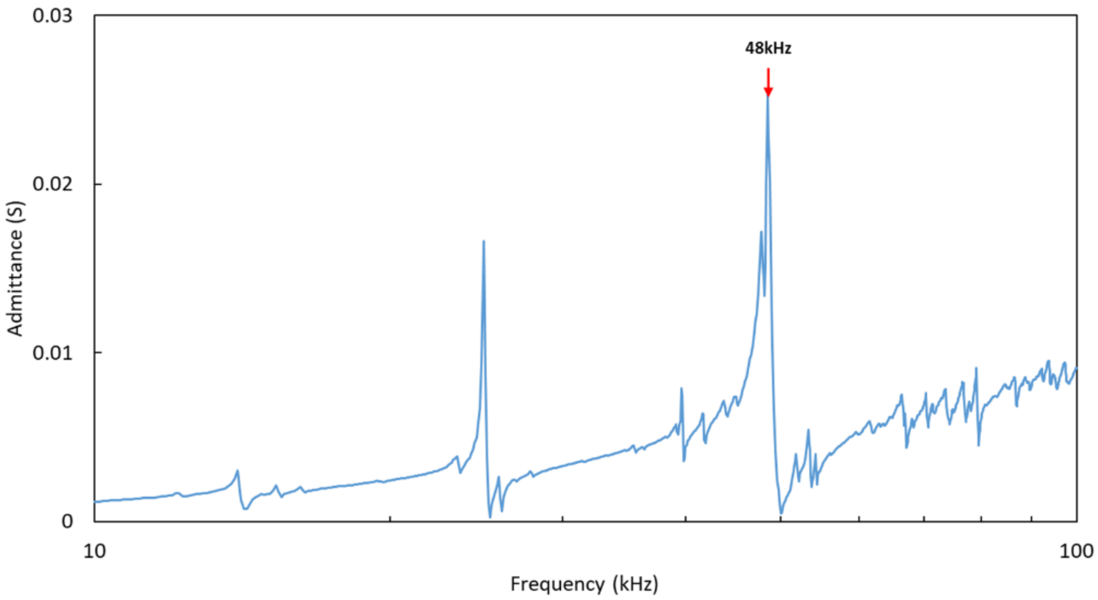

Figure S1. The admittance curve for the borosilicate glass microreactor coupled with a piezoelectric plate transducer. The resonance frequency of 48 kHz is indicated in the admittance curve.

Liquid Residence Time Distribution

The liquid residence time of the reactor was measured utilizing a pulse residence time distribution (RTD) experiment with Methylene Blue (MB) as a tracer in ethanol. The absorbance values for the tracer MB for the concentration range of 0.014mM to 0.47mM in ethanol were obtained using the spectrometer and the flow cell at the absorption wavelength of 609.9 nm. The absorbance of the tracer was linear with the MB concentration in the considered concentration range.

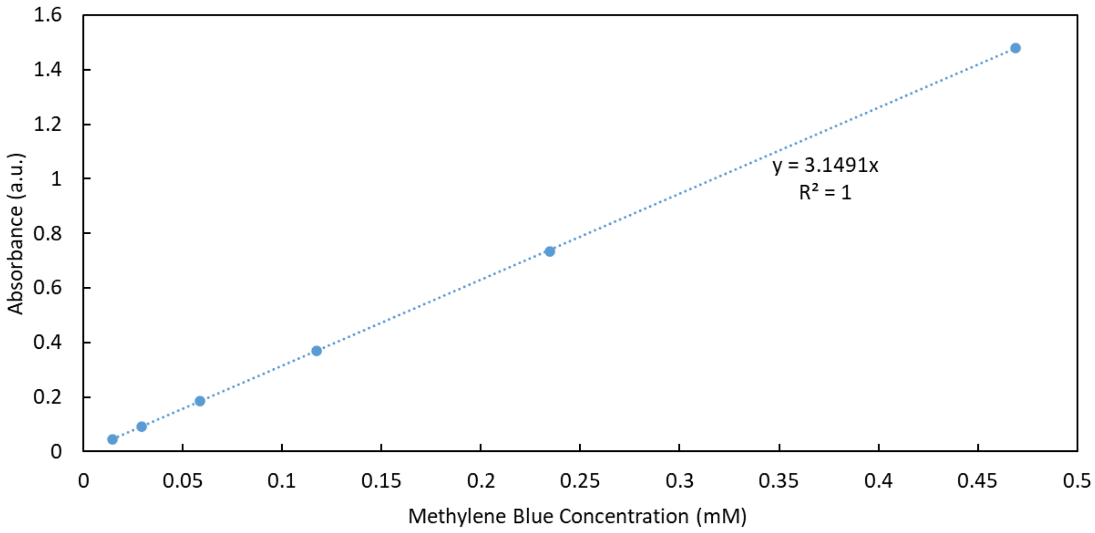

Figure S2. The absorbance vs concentration for Methylene Blue in ethanol.

The 'inlet' for the reactor was obtained by connecting the 6-way valve directly to the UV-Vis in-line flow cell. The 'outlet' for the reactor was obtained by connecting the reactor to the 6-way valve, followed by the UV-Vis in-line flow cell. For the liquid RTD experiments, the tracer MB (0.3 mg/ml, 0.94 mM) was injected into the carrier stream of ethanol via a 6-way valve. The trigger was switched to 'ON' as soon as the tracer was injected to start the data acquisition. The MB absorbance was obtained for the wavelength of 609.9 nm for an integration time of 1 ms.

The inlet residence time, i.e., the residence time in the volume of the tubing after the 6-way valve was calculated from the RTD measurement of the 'inlet' using Equation S1. The outlet residence time, i.e., the residence time in the reactor and the volume of the tubing after the 6-way valve was calculated from the RTD measurement of the 'outlet' using Equation S2. The reactor residence time in the reactor was determined from the difference between the outlet and the inlet residence time. The inlet and outlet measurements are repeated three times to obtain the reactor residence time.

$$t_{inlet} = \frac{\int_0^\infty t c_{inlet}(t)dt}{\int_0^\infty c_{inlet}(t)dt} \qquad \text{Equation S1}$$

$$t_{outlet} = \frac{\int_0^\infty t c_{outlet}(t)dt}{\int_0^\infty c_{outlet}(t)dt} \qquad \text{Equation S2}$$

$$t_{reactor} = t_{outlet} - t_{inlet}$$ Equation S3

The tracer spreads in the microchannel operating in the laminar regime due to axial dispersion. In this study, the axial dispersion model was used to determine the axial dispersion of the tracer for the silent and sonicated conditions [1]. For *D/ul* < 0.01

$$E = \sqrt{\frac{u^3}{4\pi DL}}\, exp\left[-\frac{(L-ut)^2}{4DL/u}\right]$$ Equation S4

For *D/ul* > 0.01

$$E = \frac{u}{\sqrt{4\pi Dt}}\, exp\left[-\frac{(L-ut)^2}{4Dt}\right]$$ Equation S5

where *D/ul* is the vessel dispersion number, *D* is the dispersion coefficient, *u* is the superficial liquid velocity, and *L* is the length of the microchannel. This dispersion model is appropriate for the open-open boundary condition.[1-3]

The inlet response was convoluted with the axial dispersion model to fit the vessel dispersion number and the residence time and match the outlet response. The fitting was performed by the least-square fitting method in MATLAB. The experimental residence time and the fitted residence time and the vessel dispersion number are shown in Table S1 and S2.

Table S1. Experimental residence time, model-fitted residence time, and the vessel dispersion number for the silent condition.

	Experimental Residence Time (s)	Model-Fitted Residence Time (s)	Vessel Dispersion Number (*D/uL*)
1	628.72	566.84	0.0496
2	632.04	576.18	0.0487
3	652.20	584.74	0.0456

Table S2. Experimental residence time, model-fitted residence time, and the vessel dispersion number for the sonicated condition.

	Experimental Residence Time (s)	Model-Fitted Residence Time (s)	Vessel Dispersion Number (*D/uL*)
1	619.87	601.29	0.0159
2	652.87	608.94	0.0083
3	645.07	607.58	0.0133

Solid Residence Time Distribution

The solid RTD was measured using potassium ferricyanide $K_3[Fe (CN)_6]$ as the solid tracer.[4] A potassium ferricyanide suspension in acetonitrile was prepared for a solid concentration of 9 mg/ml. The pulse RTD experiments were performed using a six-way valve for the injection of the solids. The potassium ferricyanide particles are insoluble in acetonitrile, however, are soluble in water. A stream of water is utilized to dissolve the solid particles while mixing with the water-miscible carrier phase. A water-to-acetonitrile flow rate ratio of 1:1.09 was employed for the RTD experiments to ensure the complete dissolution of the solid particles while avoiding any degassing on mixing the two streams.

A calibration curve was obtained for potassium ferricyanide in the water-acetonitrile mixture. Water and acetonitrile are mixed for a volume ratio of 100:109 for preparing the solid-water-acetonitrile mixture. The absorbance values of the solid tracer in water-acetonitrile were obtained using the spectrometer and the flow cell at the absorption wavelength of 420.2 nm. The absorbance of the tracer particles was linear with the concentration. The solid suspension was introduced in the tracer loop moments before starting the RTD experiment to avoid settling the solids in the loop. The solid RTD experiments were performed similarly to the liquid RTD experiments. The 'inlet' and the 'outlet' for the solid RTD experiment were measured similarly to the liquid RTD experiment. The average inlet and outlet RTD were obtained by averaging four repetitions of inlet and outlet experiments.

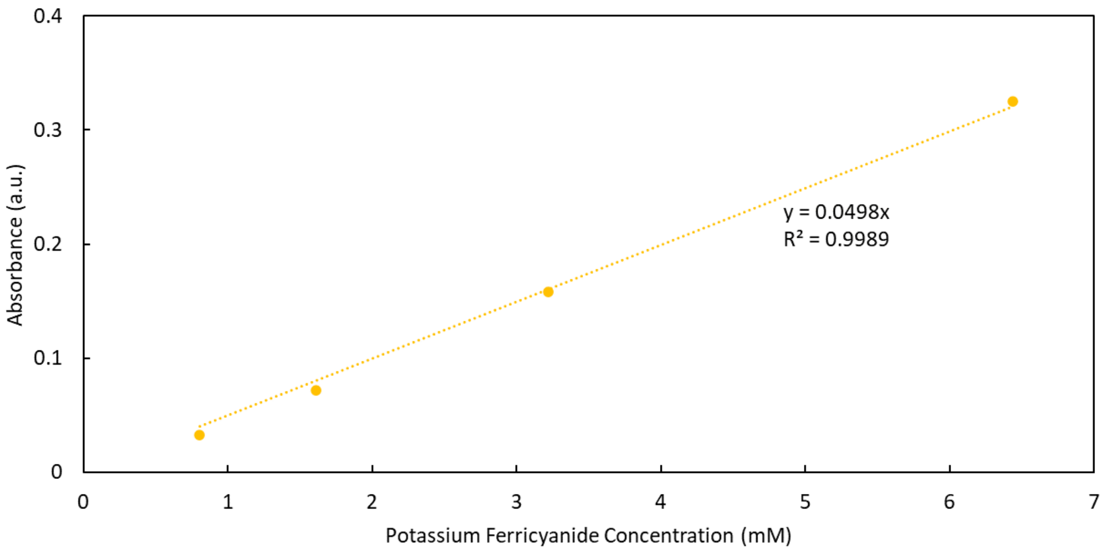

Figure S3. The absorbance vs concentration for Potassium Ferricyanide in the water-acetonitrile mixture.

The reactor residence time was obtained by utilizing Equation S1-S3. The axial dispersion model was utilized to determine the axial dispersion of the solids in the microreactor for the sonicated case. Similar to the liquid RTD, the inlet response was convoluted with the axial dispersion model to fit the vessel dispersion number and the residence time to match the outlet response.

Table S3. Experimental residence time, model-fitted residence time, and the vessel dispersion number for the solid RTD.

Experimental Residence Time (s)	Model-Fitted Residence Time (s)	Vessel Dispersion Number (*D/uL*)
669.88	606.36	0.0024

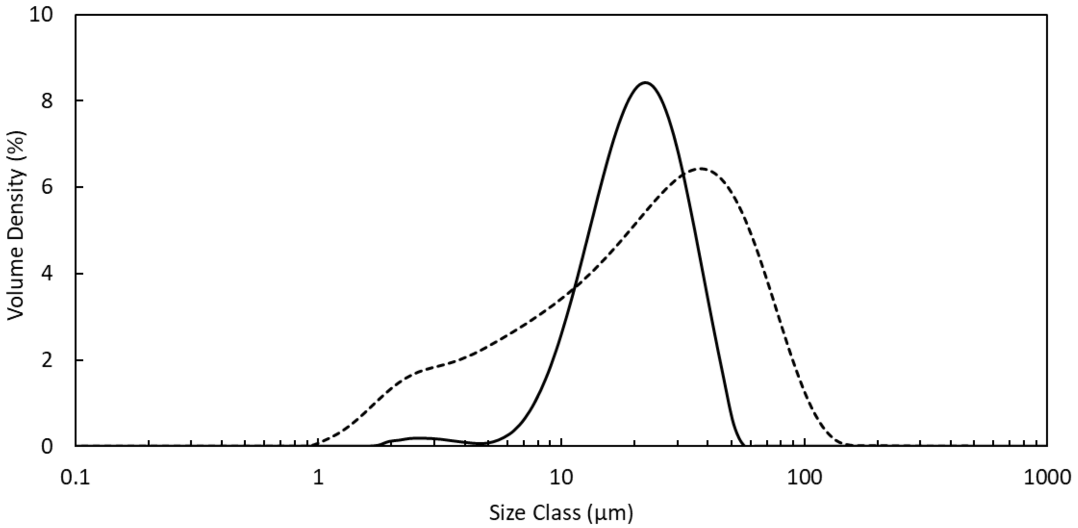

Figure S4. The particle size distribution of the Na_2CO_3 particles (-) and the tracer particles ($K_3[Fe(CN)_6]$) (--). The D_{50} for the Na_2CO_3 particles and tracer particles is 21 µm and 22.5 µm respectively. The particle size is measured using Malvern Mastersizer 3000.

The theoretical settling time for the $K_3[Fe (CN)_6]$ particles and the Na_2CO_3 particles in the reactor channel of depth 1.2 mm was calculated based on the Stokes' law (Equation S6).

$$v = \frac{d^2(\rho_s - \rho_l)g}{18\eta} \quad \text{Equation S6}$$

Table S4. The properties of the solids and liquids and the settling velocity and settling time.

Solid-Liquid	Mean Particle Diameter (d) (µm)	Particle Density (ρ_s) (kg/m^3)	Liquid Density (ρ_l) (kg/m^3)	Gravitational Constant (g) (m^2/s)	Liquid Viscosity (η) (mPa.s)	Settling Velocity (v) (mm/s)	Settling Time (s)
$K_3[Fe(CN)_6]$-Acetonitrile	22.6	1890	786	9.8	0.38	0.81	2.48
Na_2CO_3-DME	21	2540	870	9.8	0.46	0.872	1.38

Chemical Actinometry

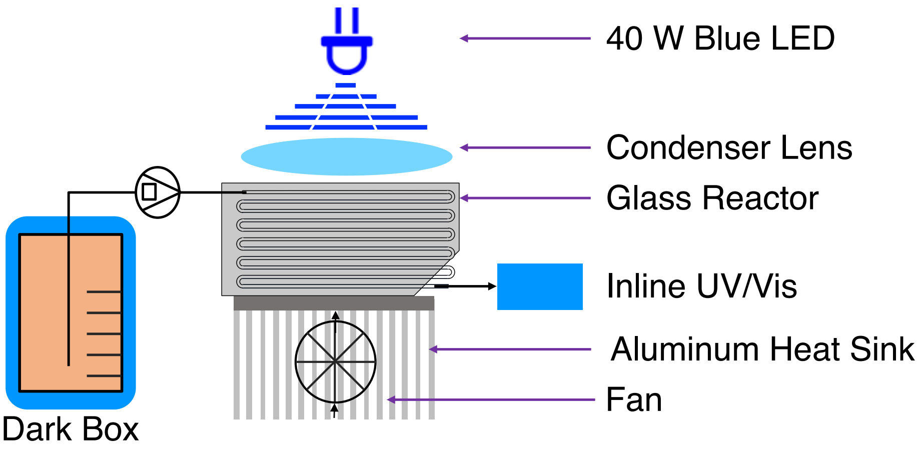

Figure S5. Schematic of setup used for the chemical actinometry.

Chemical actinometry of the microreactor was performed at different electrical input powers for a 40 W blue LED (Kessil lightening A160 WE) to measure the absorbed photon flux (Figure S5). The A-Series Spectral Controller provided by Kessil Lightening was used to adjust the electrical input power (5%, 10%, 25%, 50%, 75% and 100%) of the LED. Oxidation of 9,10-diphenylantracene (DPA, 0.5 mM) with 1.0 mM $Ru(bpy)_3Cl_2$ in acetonitrile was used as a chemical actinometer as in our previous publications.[4]

Silyl Mediated Photochemical Reaction

Pump 1 (Syringe pump - slurry): 2-bromobenzonitrile (**1**, 109 mg, 0.60 mmol, 1.0 equiv.), 4-bromotetrahydro-2H-pyran (**2**, 101 μL, 0.90 mmol, 1.5 equiv.), $Ir[dF(CF_3)ppy]_2(dtbbpy)PF_6$ (13.5 mg, 12.0 μmol, 0.02 equiv.), $NiCl_2$.glyme (2.38 mg, 6.0 μmol, 0.01 equiv.), 4,4'-di-tert-butyl-2,2'-bipyridine (ditbbpy) (1.93 mg, 7.2 μmol, 0.012 equiv.), Na_2CO_3 (127 mg, 1.2 mmol, 2.0 equiv.), (TTMSS) (194 mg, 0.78 mmol,1.3 equiv.), naphthalene (7.69 mg, 0.06 mmol, 0.1 equiv.) (internal standard for HPLC), trimethoxy benzene (10.1 mg, 0.06 mmol, 0.1 equiv.) (internal

standard for NMR), and DME (30 mL). Na_2CO_3 particles were milled and then sieved with sieve no. 200.

Pump 2 (Syringe pump): Degassed DME – connected to T-connecter at the inlet of the microreactor. This is used to prefill and clean the microreactor.

Under flow conditions: All the reagents in the slurry syringe Pump 1 are degassed. The stainless-steel syringe (Pump 1) with slurry contains a PTFE coated magnetic stir bar, which is stirred by a magnetic stirrer to keep the slurry uniformly in suspension. The flow run is initiated by prefilling the microreactor with the degassed DME (Pump 2) and DME flow rate was set to 0.1 mL/min. The microreactor was sonicated (frequency: 48 kHz, power: 5 W) and illuminated with the 40 W blue LED. The cooling fan was started to regulate the microreactor temperature. Then the DME flow (Pump 2) was stopped, and the slurry flow (Pump 1) was set at 0.1 mL/min for the residence time of 11 minutes. The flow photochemical reaction in the ultrasonic microreactor was carried out for 3 hours. The steady state (after ~ 3 residence times) sample was analyzed with HPLC (Agilent 1260). The steady state sample was purified by column chromatography (ethyl acetate:hexane = 2:8) and obtained the pure product as a white solid (analyzed by NMR).

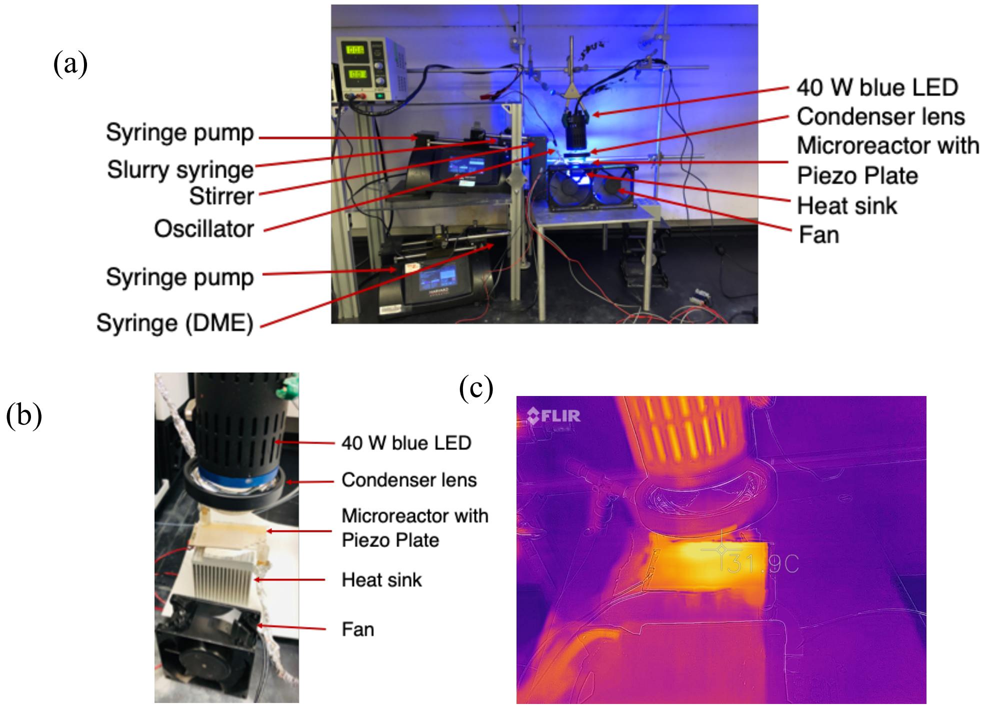

Figure S6. (a and b) Experimental setup for silyl mediated photochemical reaction, (b) FLIR ONE Pro (IR camera) image taken during silyl mediated photochemical reaction.

HPLC (Agilent 1260) method: Zorbax Bonus – RP, 4.6 X 150 mm, 3.5 μM particle size. 1.0 mL/min flow, and 25 °C column temperature, detect at 270 nm, solvent A = 1 mL/L TFA in acetonitrile, and solvent B = 1 mL/L TFA in water. Gradient elution (min): T(0) 20%A to 60%B at T(1.0), T (1.0) 40%A to 50%B at T(10.0), T(10.0) 50%A to 5%B at T(12.0), T(12.0) 95%A to 95%B(T14.0), T (14.0) 5%A to 50%B at T (15.0), 16 min run time.

NMR: 1H NMR (400 MHz, $CDCl_3$) δ 7.70 – 7.53 (m, 2H), 7.39 (d, *J* = 7.9 Hz, 1H), 7.32 (td, *J* = 7.6, 1.2 Hz, 1H), 4.15 – 4.06 (m, 2H), 3.60 (td, *J* = 11.4, 3.2 Hz, 2H), 3.25 (tt, J = 10.7, 5.2 HZ, 1H), 1.92 – 1.76 (m, 4H).

NMR data:

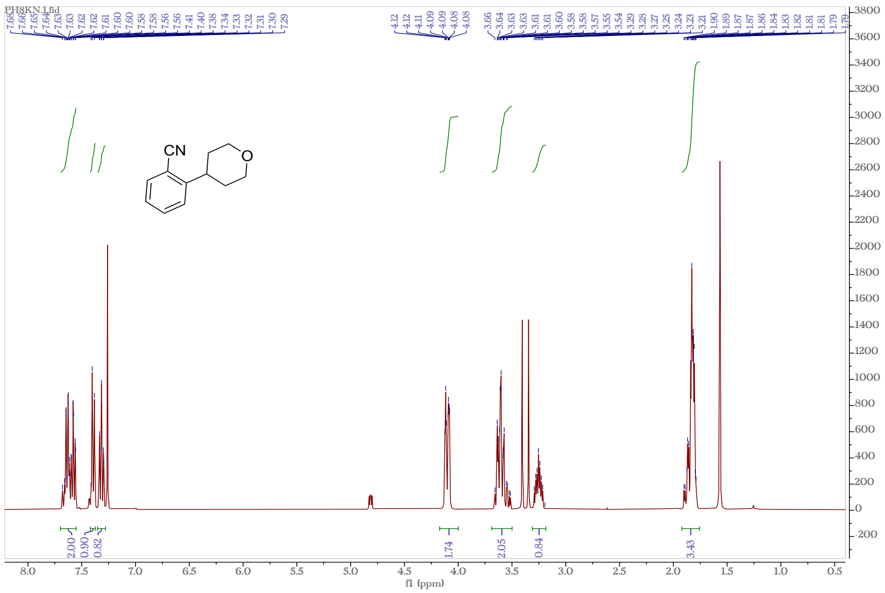

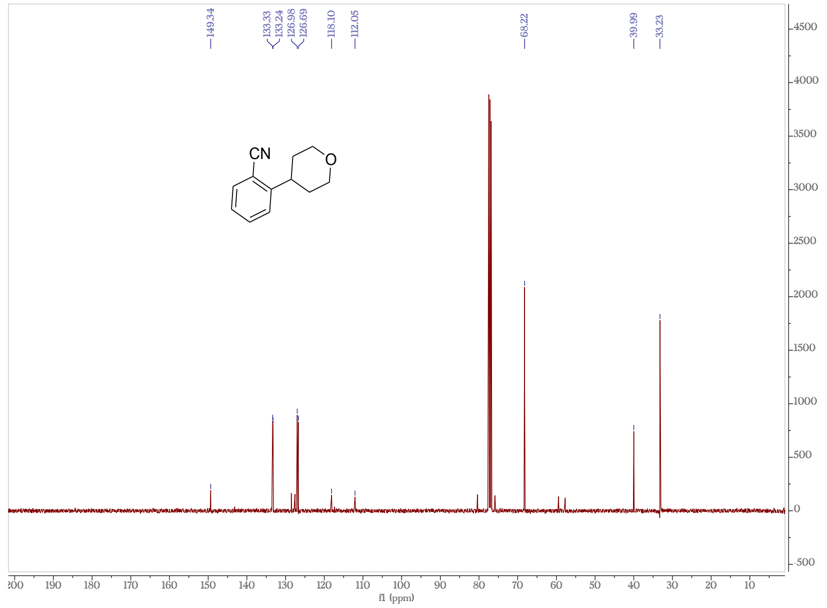

13C NMR (101 MHz, $CDCl_3$) δ 149.34, 133.33, 133.24, 126.99, 126.70, 118.10, 112.05, 68.22, 39.99, 33.24.

References

1. O. Levenspiel, *Ind Eng Chem Res*, 1999, **38**, 4140-4143.
2. P. V. Danckwerts, *Chem Eng Sci*, 1953, **2**, 1-13.
3. A. Roibu, T. Van Gerven and S. Kuhn, *ChemPhotoChem*, 2020, **4**, 5181-5192.
4. A. Pomberger, Y. Mo, K. Y. Nandiwale, V. L. Schultz, R. Duvadie, R. I. Robinson, E. I. Altinoglu and K. F. Jensen, *Organic Process Research & Development*, 2019, **23**, 2699-2706.
